# Selective Retina Therapy in Patients With Chronic Central Serous Chorioretinopathy: A Pilot Study: Erratum

**DOI:** 10.1097/01.md.0000481967.87505.c9

**Published:** 2016-03-11

In the article “Selective Retina Therapy in Patients With Chronic Central Serous Chorioretinopathy: A Pilot Study”,^1^ which appeared in Volume 95, Issue 3 of *Medicine*, Dr. Theisen-Kunde's and Dr. Brinkmann's names were originally presented incorrectly. The article has since been corrected online.
